# A Simulation Study of the Use of Vaccination to Control Foot-and-Mouth Disease Outbreaks Across Australia

**DOI:** 10.3389/fvets.2021.648003

**Published:** 2021-08-11

**Authors:** Tim R. Capon, Michael G. Garner, Sorada Tapsuwan, Sharon Roche, Andrew C. Breed, Shuang Liu, Corissa Miller, Richard Bradhurst, Sam Hamilton

**Affiliations:** ^1^CSIRO Land & Water, Acton, ACT, Australia; ^2^CSIRO Health & Biosecurity, Acton, ACT, Australia; ^3^Epidemiology and One Health Section, Department of Agriculture, Water and the Environment, Canberra, ACT, Australia; ^4^School of Veterinary Science, University of Queensland, Brisbane, QLD, Australia; ^5^Centre of Excellence for Biosecurity Risk Analysis, The University of Melbourne, Melbourne, VIC, Australia

**Keywords:** Australian animal disease spread model, AADIS, vaccination, stamping out, epidemiology, outbreak, livestock

## Abstract

This study examines the potential for foot-and-mouth disease (FMD) control strategies that incorporate vaccination to manage FMD spread for a range of incursion scenarios across Australia. Stakeholder consultation was used to formulate control strategies and incursion scenarios to ensure relevance to the diverse range of Australian livestock production regions and management systems. The Australian Animal Disease Spread model (AADIS) was used to compare nine control strategies for 13 incursion scenarios, including seven control strategies incorporating vaccination. The control strategies with vaccination differed in terms of their approaches for targeting areas and species. These strategies are compared with two benchmark strategies based on stamping out only. Outbreak size and duration were compared in terms of the total number of infected premises, the duration of the control stage of an FMD outbreak, and the number of vaccinated animals. The three key findings from this analysis are as follows: (1) smaller outbreaks can be effectively managed by stamping out without vaccination, (2) the size and duration of larger outbreaks can be significantly reduced when vaccination is used, and (3) different vaccination strategies produced similar reductions in the size and duration of an outbreak, but the number of animals vaccinated varied. Under current international standards for regaining FMD-free status, vaccinated animals need to be removed from the population at the end of the outbreak to minimize trade impacts. We have shown that selective, targeted vaccination strategies could achieve effective FMD control while significantly reducing the number of animals vaccinated.

## Introduction

Foot-and-mouth disease (FMD) is recognized as the single greatest disease threat to Australia's livestock industries (1, 2). Early detection of an incursion, effective control of an outbreak, and rapid return to trade are essential to minimize the economic impact of an outbreak. Australia's policy for an FMD response is to contain, control, and eradicate the disease and re-establish the FMD-free status of Australia as quickly as possible, while minimizing social and financial disruption. The Australian Veterinary Emergency Plan (AUSVETPLAN) states that the “re-establishment of trade for affected industries would be one of the highest priorities of disease response efforts” (3).

Australia's preferred approach to control an outbreak of FMD is to use stamping out, supported by a combination of measures that include a national livestock standstill, quarantine, regional movement controls, tracing, and surveillance (3). Additional measures that may be taken if authorities consider that they would be beneficial in containing and managing the outbreak include vaccination, pre-emptive culling, zoning/compartmentalization, and risk-based movement controls. Australia invests considerable resources in preparedness and planning for emergency animal diseases, including maintaining a government- and industry-funded vaccine bank for FMD (3). Despite changes to Australian contingency plans to recognize that vaccination could be an important component of an FMD control program as soon as an outbreak is detected, it is unclear how, when, or even if vaccination should be used, and if it is used, how vaccinated animals should be managed.

Modeling studies in Australia (4–6) and overseas (7–9) have shown that vaccination is effective in reducing the duration and size of outbreak situations where disease is widespread, where there is a high rate of spread, or resources for stamping out are limited. Reports suggest that early vaccination may have allowed earlier eradication that took place in FMD outbreaks in Korea (10) and Japan (11, 12). Thus, vaccination is increasingly recognized as a useful tool in containing and eradicating FMD outbreaks. However, while vaccination can contribute to earlier eradication of disease, it will have additional costs—keeping vaccinated animals in the population will delay the period until FMD-free status is regained under the World Organization for Animal Health standards (13)—and add additional complexity to the post-outbreak surveillance for demonstrating the re-establishment of FMD-free status. These issues are of particular concern for countries with significant exports of livestock and livestock products as the use of vaccination and the presence of FMD vaccinated animals in the population could be expected to cause significant market access difficulties.

Australia has no recent experience with controlling an outbreak of FMD. Decision support tools including disease models offer valuable insights into the effectiveness of different control measures (14). In particular, the decision to vaccinate is best made early in an outbreak as vaccination is likely to perform better when implemented earlier (5). However, a decision to vaccinate early in the outbreak may result in using vaccination in situations where it may offer little to no additional benefit with implications for post-outbreak surveillance, management of vaccinated animals, and regaining FMD-free status and access to markets. Conversely, not using vaccination may lead to larger and longer outbreaks, increased control costs and greater ongoing impacts on industry and local communities.

While a number of modeling studies have already assessed FMD spread and control in Australia [e.g., (4–6)], these have tended to focus on a limited range of introduction scenarios along the eastern seaboard, representing scenarios considered to be most likely or worst-case situations for FMD introduction and spread. FMD introduction, spread, and control in other areas of Australia are poorly understood. Disease managers would benefit from a clearer understanding of how, and under what conditions, vaccination could provide benefits in terms of managing an FMD outbreak in Australia.

The objective of this study is to thoroughly investigate the possible incursion scenarios and control options available to manage an FMD outbreak, with a focus on vaccination as a disease control option. The first stage of this study elicited stakeholders' views regarding the use of vaccination as part of a control strategy, incursion scenarios, and factors affecting emergency animal disease management decisions. The second stage of the study focused on how vaccination might be applied and the effect of vaccination on the size and duration of an outbreak. Drawing on the results of the stakeholder consultations, simulations were designed to better understand the consequences of alternative approaches to incorporating vaccination into control strategies for FMD.

## Methods

### Stakeholder Consultation

Inputs from Australian state and territory jurisdictional stakeholders were collected through workshops and surveys. These were conducted during April to August 2017. This research received ethics approval from the CSIRO Human Ethics research committee. Stakeholders were selected from a panel of government and industry stakeholders affiliated with Animal Health Australia (AHA). AHA is a not-for-profit public company with membership made up of Commonwealth, state and territory governments, livestock industries, service providers, and associate members. AHA manages a range of national programs on behalf of its members that improve animal and associated human health, biosecurity, market access, livestock welfare, productivity, and food safety and quality (15). Selected stakeholders were sent an email invitation by AHA to participate in the consultation process (workshops or surveys depending on their availability). Two face-to-face workshops were held, each consisting of ~30 participants. Surveys were sent to representatives of the Australian jurisdictional governments to request information about two or three incursion scenarios of interest based on the most likely or important scenarios for FMD introduction for their jurisdiction. Details are provided in the Supplementary Materials.

### Simulation Study Design

Simulations were conducted using the Australian Animal Disease Spread model (AADIS) (16)^1^. AADIS is a stochastic spatial simulation model that simulates livestock disease spread and control at the national scale. AADIS uses the herd as its epidemiological unit of interest. A “herd” in AADIS is defined as a group of comingling animals of the same species under the same production system. There are 11 different herd types in the AADIS FMD model (Table 1), and this allows for common attributes such as movement patterns and biosecurity practices to be applied based on herd type.

**Table 1:** Farm and animal populations used in the AADIS FMD model.

**Farm type**	**Number of farms**	**Number of animals mean (min–max)**
Extensive beef	1,331	1,909 (1,200–46,575)
Intensive beef	51,383	280 (30–7,436)
Feedlot	508	1,825 (100–39,963)
Mixed beef/sheep	21,556	242 (30–5,700)
Dairy	8,675	298 (40–2,742)
Small pigs	1,873	244 (40–4,850)
Large pigs	333	4,922 (1,000–17,896)
Sheep	22,150	1,649 (20–44,000)
Smallholder	103,641	5 (1–14)
**Total**	**202,775**	

*This represents a synthetic farm population dataset obtained from Agricultural Census data (17) and industry data and reports. The bold values are statistically significant values*.

AADIS has a hybrid architecture that combines equation- and agent-based modeling techniques. The spread of disease within a herd is represented by an SEIR compartmental equation-based model (EBM) implemented as a system of ordinary differential equations (ODEs). The parameterization of the ODE system reflects the herd's production system and the subject FMD virus strain. At the time of infection, the herd's ODE system is solved numerically to yield predictions of the proportion of the population that are infected, infectious, and have clinical signs of disease over time. The solution remains in place until an external event such as vaccination or culling acts upon the herd, triggering the resolving of the ODE system. The spread of disease between herds is modeled with a stochastic and spatially explicit agent-based approach. The model incorporates the attributes and spatial locations of individual farms, saleyards, weather stations, local government areas, and direct and indirect movement patterns. AADIS simulates disease spread in daily time steps, and FMD transmission between herds is modeled through five discrete pathways: 1—farm to farm animal movements, 2—local spread (infection of farms and herds within close geographical proximity by unspecified means), 3—indirect contact (*via* fomites or animal products), 4—animal movements *via* saleyards or markets, and 5—wind-borne spread. The proportions of infected and infectious animals in the population predicted by a herd's EBM inform the likelihood that between-herd spread will occur.

The AADIS unit of interest for the control of disease is the “farm” — defined as an establishment that has one or more herds. AADIS simulates disease control according to the availability of resources, such as personnel and vaccine, and models the suite of control measures prescribed in AUSVETPLAN (3). These control measures include movement controls of animals and fomites (national livestock standstill, regional movement restrictions, and quarantine of farms), stamping out of different farm types (culling and disposal of animals and decontamination of farms), surveillance (farmer reporting and active surveillance within declared areas), tracing (direct and indirect contacts), pre-emptive culling (dangerous contacts, ring culling, and slaughter on suspicion), and vaccination (suppressive, protective, or mass vaccination). All control measures are defined and resourced per jurisdiction. Further details on AADIS can be found in Bradhurst et al. (16, 18).

To characterize the incursion scenarios and control strategies for this study, AADIS was parameterized using a combination of values estimated for previous studies (5) and values estimated through stakeholder consultation (as described in Section Stakeholder Consultation above). Details of the AADIS parameterization are provided in the Supplementary Materials.

### Incursion Scenarios

To examine the effectiveness of alternative approaches to incorporating vaccination into a control strategy across a range of starting conditions, we simulated control strategies for 13 incursion scenarios. Findings from the workshops and surveys were used to develop the characteristics of the incursion scenarios of interest to stakeholders. This included the method of FMD introduction, when FMD was introduced, type of source farm, time until first detection, and the reasons for selection of the scenarios. This approach ensured that the modeled outbreaks were relevant to the state and territory governments.

**Figure 1:**
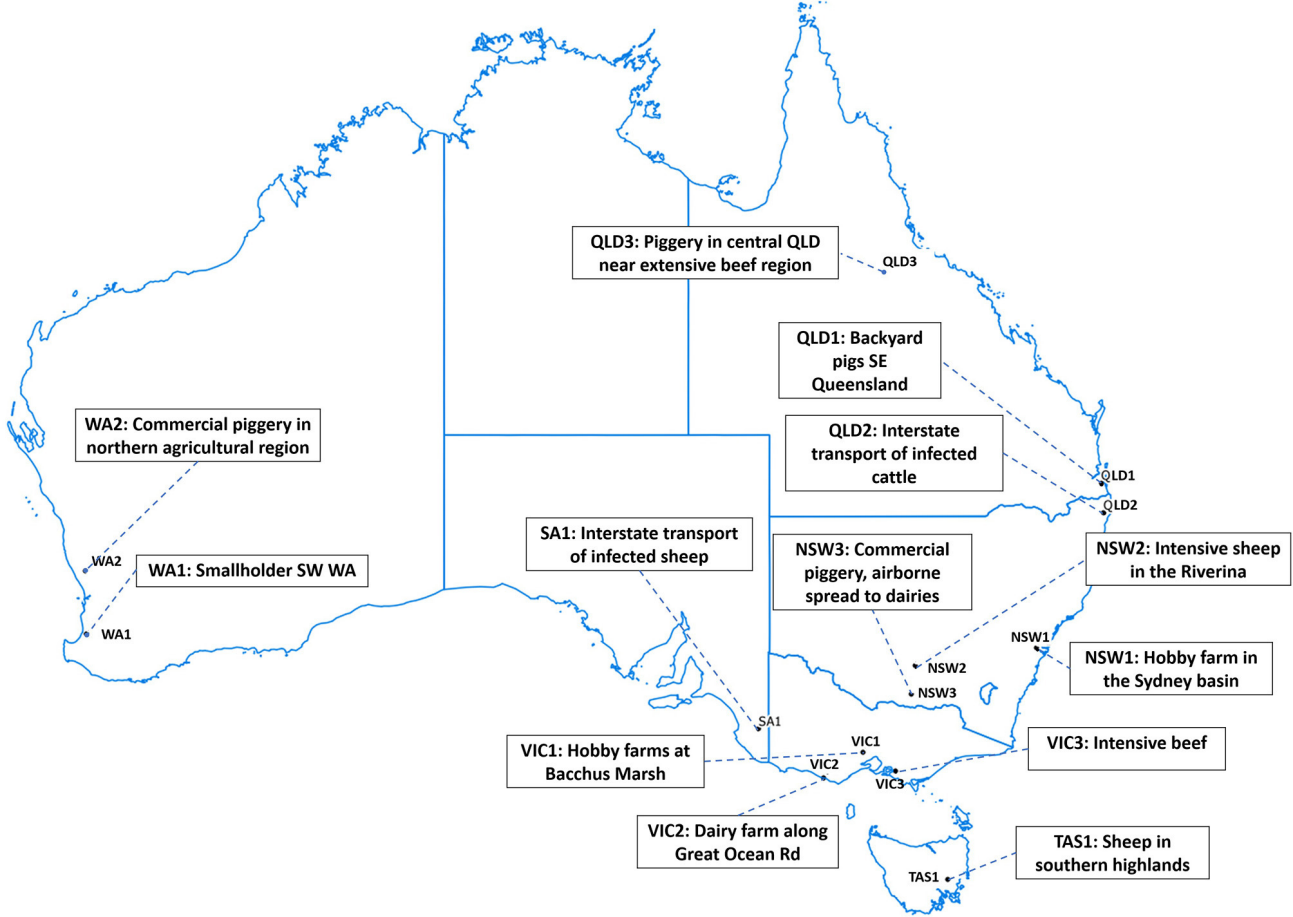
Locations of seed herds for each incursion scenario.

To convert inputs from stakeholder consultation into scenarios for the simulation study, we selected simulation runs based on stakeholders' scenario descriptions. A small set of up to 50 simulation runs was conducted for each incursion scenario at a time of year consistent with the scenario descriptions (as shown in Table 2). The simulation run that most resembled the description was used to identify the first infected farm, or “seed herd,” for each incursion scenario. The selection was based on species, farm type, and geography of the starting location. Time until detection was fixed across incursion scenarios to focus comparisons on differences due to geographical conditions. A time of 21 days of silent spread before detection and disease control begins was chosen based on recent studies in Australia (6, 19, 20). The disease situation at detection (i.e., at the end of the silent spread phase of these representative runs) was saved as a “snapshot.” Figure 1 shows the locations of the seed herds for each of the 13 incursion scenarios.

**Table 2:** Starting conditions for simulation study FMD incursion scenarios: seed herds and snapshots.

**Incursion scenario**	**ID**	**Scenario description**	**Seed herd**			**Snapshot**
			**Scenario starting date**	**Farm type**	**# animals**	**# infected herds when FMD is first detected** ^**a**^
New South Wales	NSW1	Hobby farm in the Sydney basin	May 10	Smallholder	8	6
	NSW2	Intensive sheep in the Riverina	November 10	Sheep farm	1,210	2
	NSW3	Commercial piggery, airborne spread to dairies	July 1	Commercial piggery	4,643	9
Queensland	QLD1	Backyard pigs in South Eastern Queensland	January 10	Smallholder	9	4
	QLD2	Interstate transport of infected cattle	June 10	Intensive beef	109	13
	QLD3	Piggery in central Queensland near extensive beef region	May 1	Small pig farm	363	2
South Australia	SA1	Interstate transport of infected sheep	November 1	Mixed sheep/beef	3,271	3
Tasmania	TAS1	Sheep in southern highlands	August 10	Sheep farm	1,418	2
Victoria	VIC1	Hobby farms at Bacchus Marsh	May 1	Smallholder	12	3
	VIC2	Dairy farm in South Western Victoria	September 10	Dairy herd	516	44
	VIC3	Intensive beef in South East Victoria	October 1	Intensive beef	89	16
Western Australia	WA1	Smallholder in South West WA	May 10	Smallholder	7	10
	WA2	Commercial piggery in northern agricultural region	May 10	Commercial piggery	10,836	10

a *Simulated number of infected herds in the population when the first IP is confirmed at the end of the silent spread phase*.

The use of snapshots to capture the details of the incursion scenarios in AADIS ensured that alternative control strategies could be compared from an identical starting point when the disease was first detected, and control commenced.

### Design of Control Strategies

For this study, nine control strategies were selected to provide a comparison of seven alternative approaches using vaccination with two benchmark control strategies with stamping out but no vaccination. Stamping out is the default approach for controlling an outbreak of FMD and aims to ensure infected premises are quarantined and that susceptible animals are destroyed to limit virus spread (3). For each incursion scenario, 500 simulation runs were conducted of each control strategy. Preliminary work has shown that this is adequate in providing a high degree of convergence (<5%) for key outbreak metrics (number of IPs, duration, and costs). Convergence provides an indication across a set of simulation runs to how close the sample mean of key “per-run indicators” is to the theoretical population mean (21).

Table 3 describes the main points of difference between the control strategies.

**Table 3:** Description of each control strategy in terms of approach to stamping out and targeting of vaccination.

**Control strategy**	**Stamping out**	**Pre-emptive culling of DCPs^**%**^**	**Vaccination**	**Targeting of vaccination**		
				**Animals/Operations**	**Ring or Annulus**	**Area**
1	Yes	No	No	–	–	–
2	Yes	Yes	No	–	–	–
3	Yes	No	Yes	All species^*^	5-km ring	All
4	Yes	No	Yes	All species	5-km ring	High-risk area^#^
5	Yes	No	Yes	All species except pigs and smallholders	5-km ring	All
6	Yes	No	Yes	Vaccination of specialist cattle producers^∧^	5-km ring	All
7	Yes	No	Yes	Vaccination of specialist cattle producers^∧^	5-km annulus, 5-km from IPs (out–in)	High-risk area^#^
8	Yes	No	Yes	Feedlots and large dairy farms >500 head	5-km annulus, 5-km from IPs (out–in)	All
9	Yes	Yes	Yes	All species	5-km ring	All

* *Beef cattle on extensive properties were not targeted for vaccination in any control strategy because large extensive cattle properties are found only in northern Australia. They involve large areas with very low stocking densities and they are considered a low risk for FMD establishing/spreading*.

∧ *Including feedlots, dairy and intensive beef farms, but excluding extensive beef and mixed beef–sheep farms to avoid including large numbers of sheep on mixed farms in the vaccination program*.

# *High-risk areas were defined as local government areas with high cattle herd densities and high cattle densities (>25 cattle per sq km). ^%^DCPs are “Dangerous Contact Premises”*.

All control strategies included stamping out. Control Strategies 2 and 9 included the pre-emptive culling of DCPs. Control Strategies 3 to 9 included some form of vaccination in addition to stamping out. For all vaccination strategies, vaccination was triggered on day 14 of the control phase only if there were five or more infected premises (IPs), as it was considered unlikely that vaccination would be applied if there were only a small number of IPs. The approaches to vaccination differed in terms of the animal species and farm types targeted, whether a suppressive vaccination approach was used (5-km radius ring around an IP with vaccination from inside out) or a protective vaccination approach within an annulus (5 km wide starting 5 km away from an IP, i.e., in an area between 5 and 10 km from the IP, with vaccination occurring from the outside in) was used, and whether all areas were targeted or only herds in pre-identified high-risk, livestock-dense areas were targeted for vaccination. High-risk areas were defined as local government areas with high cattle herd density (>0.175 herds per sq km) and high cattle density (>25 cattle per sq km). Estimates of resource teams available to undertake control activities were provided by jurisdictional animal health staff and considered the availability of resources from both the public and private sectors. Details of the model settings and parameters are included in the Supplementary Materials.

### Sensitivity Analysis

In addition to the baseline control strategies, sensitivity analyses were conducted to test how sensitive the results are to two key assumptions used in the study:

- Timing of vaccination—vaccination was assumed to start 14 days into the control program based on the expected time for vaccine to be available for deployment. To test the sensitivity of the results to timing of vaccination, vaccination programs starting on day 10 and day 21 were also simulated.
- Vaccination ring radius—based on stakeholder inputs, we assumed a 5-km ring vaccination radius. To test how sensitive the results are to the size of the vaccination ring, we also simulated a 3-km ring radius.

For the sensitivity analyses, the nine control strategies were run for each of the 13 incursion scenarios, with changed parameter values for these assumptions. Previous studies have conducted sensitivity analyses of other AADIS parameters, including time to detection and duration of the national standstill (16), and parameters relevant to FMD transmission, such as the probability of spread, infectivity, and susceptibility (22).

### Statistical Analysis

The Kruskal–Wallis test was used to test whether there were differences in the mean number of IPs and the last day of control for each incursion scenario and control strategy combination. This test is a non-parametric analog to the ANOVA and was chosen as the appropriate test due to the data being non-normally distributed. The null hypothesis is that there are no significant differences in the median number of IPs and the last day of control for each of the control strategies, for each of the starting locations. The null hypothesis was rejected at the 95% confidence level. To examine specifically which control strategies and which incursion scenarios result in significant differences in the number of IPs and last day of control, we performed a Dunn test (23). The Dunn test is the appropriate non-parametric pairwise multiple comparison procedure when a Kruskal–Wallis test is rejected (24). We applied a Bonferroni adjustment to account for the number of pairwise comparisons conducted.

## Results

We compared alternative disease control strategies that incorporate vaccination with benchmark control strategies with stamping out only, across the range of incursion scenarios. We first present the results for the benchmark strategies, then the assessment of the effectiveness of vaccination based on a comprehensive ring vaccination approach (Control Strategy 3) for all incursion scenarios, before providing a more detailed analysis of the alternative types of vaccination strategy. Finally, we report the results of the sensitivity analyses.

### Incursion Scenarios Derived From Stakeholder Consultation

Table 2 describes the starting conditions of each incursion scenario, including production type and number of animals in each seed herd and the number of infected herds in the population for each snapshot, that is, when the outbreak is first detected, and the control program begins.

### Benchmark Strategies for All Incursion Scenarios

For comparison with alternative approaches using vaccination, simulations were conducted with a benchmark strategy of stamping out only (Control Strategy 1) and stamping out with pre-emptive culling of DCPs (Control Strategy 2). Tables 4, 5 present descriptive statistics for all incursion scenarios for the benchmark, Control Strategy 1, for the total number of IPs and the last day of control (i.e., duration of the control program).

**Table 4:** Descriptive statistics for the Control Strategy 1 benchmark control strategy for all incursion scenarios.

**Variable**	**Scenario**	**Mean**	**SD**	**Min**	**Max**	**p25**	**p50**	**p75**	**p95**
Total number of IPs	NSW1	10	3	6	38	8	9	11	15
	NSW2	2	0	2	4	2	2	3	3
	NSW3	12	4	9	62	10	11	12	17
	QLD1	5	1	4	16	4	5	5	8
	QLD2	36	8	19	73	31	36	41	49
	QLD3	2	6	1	123	1	2	2	3
	SA1	5	1	3	17	5	5	6	7
	TAS1	2	1	2	5	2	2	3	4
	VIC1	2	0	2	3	2	2	2	2
	VIC2	872	690	218	5,593	528	734	1,046	1,511
	VIC3	128	225	30	3,291	72	91	116	226
	WA1	23	9	11	82	18	21	26	42
	WA2	15	3	10	63	13	14	15	18
Last day of control	NSW1	47	5	41	80	43	46	48	57
	NSW2	41	3	40	71	40	40	40	46
	NSW3	51	6	45	93	48	49	51	63
	QLD1	48	3	43	74	46	48	49	53
	QLD2	62	12	48	137	54	57	65	87
	QLD3	36	21	28	356	29	32	40	49
	SA1	48	6	44	86	45	45	49	63
	TAS1	47	3	42	75	45	46	48	51
	VIC1	39	0	39	40	39	39	39	39
	VIC2	223	82	112	718	175	207	249	348
	VIC3	124	61	59	609	92	109	136	201
	WA1	64	21	44	195	55	58	64	107
	WA2	51	4	45	83	48	50	52	60

**Table 5:** Descriptive statistics for Control Strategy 3 for all incursion scenarios.

**Variable**	**Scenario**	**Mean**	**SD**	**Min**	**Max**	**p25**	**p50**	**p75**	**p95**
Total number of IPs	NSW1	10	2	6	22	8	9	11	14
	NSW2	2	0	2	4	2	2	2	3
	NSW3	12	3	9	52	10	11	12	16
	QLD1	5	1	4	19	4	5	5	7
	QLD2	36	7	19	61	31	35	40	49
	QLD3	2	6	1	127	1	2	2	3
	SA1	5	2	3	30	5	5	6	7
	TAS1	2	1	2	10	2	2	3	4
	VIC1	2	0	2	3	2	2	2	2
	VIC2	221	52	127	807	191	214	243	292
	VIC3	64	16	35	176	54	61	71	94
	WA1	21	5	11	45	17	20	24	31
	WA2	15	3	11	53	13	14	15	19
Last day of control	NSW1	48	7	40	96	43	46	49	62
	NSW2	40	2	40	55	40	40	40	43
	NSW3	56	9	45	96	49	53	61	70
	QLD1	49	4	43	78	46	49	50	55
	QLD2	63	6	46	91	61	63	65	72
	QLD3	35	15	28	321	29	33	39	47
	SA1	48	7	44	87	45	45	49	65
	TAS1	47	4	42	90	46	47	48	51
	VIC1	39	1	39	50	39	39	39	39
	VIC2	99	17	73	225	90	96	106	125
	VIC3	72	11	56	144	65	68	77	95
	WA1	62	9	47	110	56	62	66	77
	WA2	54	8	45	93	48	52	60	68

The last day of control measures the number of days of disease control as the number of days of culling plus two incubation periods (28 days). For many of the incursion scenarios, the outbreaks were small and controlled relatively quickly. The Victorian scenarios VIC2 and VIC3 were the largest, followed by WA1, a scenario in Western Australia. In particular, the VIC2 outbreak could become very large and potentially last more than 12 months.

The pre-emptive culling of DCPs is an additional control measure that could be considered to help contain and manage the outbreak. In this study, Control Strategy 2 allows comparison with Control Strategy 9, which combines vaccination with the pre-emptive culling of DCPs.

Comparing the strategies of stamping out only (Control Strategy 1) and stamping out with pre-emptive culling of DCPs (Control Strategy 2) using Dunn tests, we found that there were statistically significant differences in the total number of IPs for incursion scenarios NSW1, WA1, and WA2 and in the last day of control for NSW1 and QLD2. The differences between the medians, however, are small and do not appear important for disease control. Notably, no statistically significant differences were found between Control Strategy 1 and Control Strategy 2 for the two incursion scenarios with the largest outbreaks, i.e., VIC2 and VIC3.

### The Effect of Vaccination on Outbreak Size and Duration

**Figure 2:**
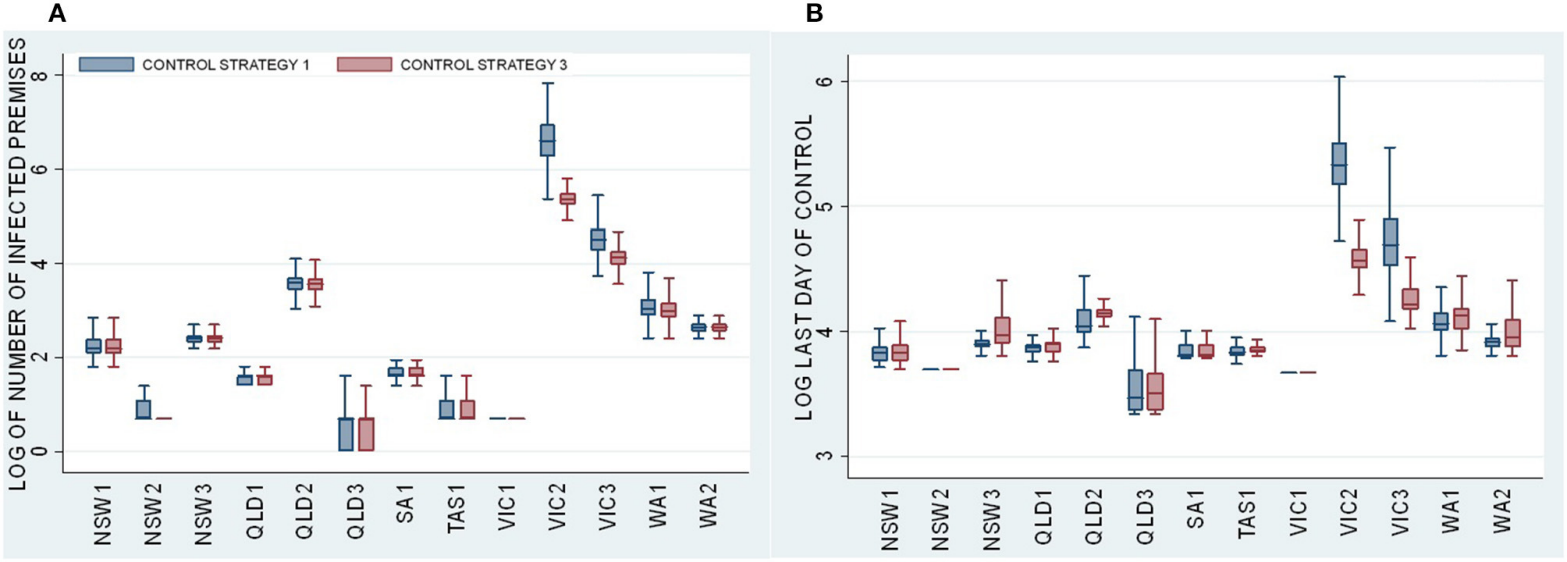
Boxplots of **(A)** the log of total number of IPs and **(B)** the log of last day of control for the Control Strategy 1 stamping out strategy (blue) and the Control Strategy 3 vaccination strategy (red) for each incursion scenario.

Descriptive statistics and Dunn test statistics were used to compare the effect of the vaccination strategy across all 13 incursion scenarios by comparing a stamping out only (Control Strategy 1) with a comprehensive vaccination strategy (Control Strategy 3), which involves vaccinating all species in a 5-km radius of each infected premises. Figure 2 presents boxplots of the distributions of (a) the total number of IPs and (b) the last day of control across all 500 iterations for Control Strategies 1 and 3. For each incursion scenario, NSW1 to WA3, Figure 2 shows boxplots for Control Strategy 1 on the left and Control Strategy 3 on the right.

Figure 2 presents the log of the size and duration of all the outbreaks. Most of the incursion scenarios shown in Figure 2 lead to small outbreaks that are controlled relatively quickly by Control Strategy 1. Vaccination (Control Strategy 3) offers no benefits in terms of reducing the size of the outbreak (number of IPs) or duration. Note that there is very little difference in the size and duration of the smaller outbreaks (NSW1–NSW3, QLD1–QLD3, SA1, TAS1, VIC1, WA1, and WA2) between Control Strategy 1—stamping out only (blue) and Control Strategy 3—vaccination strategy (red). A Dunn test statistic confirms that there are no significant differences in the median value of the total number of IPs and last day of control between Control Strategy 1 and Control Strategy 3 for all incursion scenarios shown in Figure 2.

However, in the case of Victorian scenarios (VIC2 and VIC3), the outbreaks are larger. In these cases, vaccination is effective in reducing the size and duration of the outbreaks. There is a marked contrast between the median of 734 for the total number of IPs for the VIC2-Control Strategy 1 (stamping out only) and the median of 214 for the VIC2-Control Strategy 3 (stamping out with vaccination). The same pattern holds for VIC3, with a median of 91 IPs for VIC3-Control Strategy 1 compared with 61 for VIC3-Control Strategy 3. Although only Control Strategy 3 is presented in Figure 2, Dunn tests comparing every vaccination strategy (Control Strategy 3 to Control Strategy 9) with Control Strategy 1 showed similar effects (see Tables 6, 7).

**Table 6:** Dunn tests on number of IPs.

**Number of IPs**		**Comparisons between Control Strategies 2 to 9 and Control Strategy 1 (stamping out only)**							
**Incursion scenario**	**Dunn test statistics**	**1 vs. 2**	**1 vs. 3**	**1 vs. 4**	**1 vs. 5**	**1 vs. 6**	**1 vs. 7**	**1 vs. 8**	**1 vs. 9**
NSW1	Statistics	3.9634^***^	0.2847	−0.5694	−0.0694	−0.3014	0.0407	−0.8018	5.4542^***^
NSW1	*p*-value	**0.0013**	1.0000	1.0000	1.0000	1.0000	1.0000	1.0000	**0.0000**
NSW2	Statistics	−0.2444	1.4432	0.3430	0.7574	0.4186	1.4809	0.8098	0.4711
NSW2	*p*-value	1.0000	1.0000	1.0000	1.0000	1.0000	1.0000	1.0000	1.0000
NSW3	Statistics	−0.4152	−0.8427	−0.1364	−0.2337	0.7346	−0.1541	−0.7765	−0.6992
NSW3	*p*-value	1.0000	1.0000	1.0000	1.0000	1.0000	1.0000	1.0000	1.0000
QLD1	Statistics	−0.5503	0.0942	0.3700	−0.3724	−0.4173	−0.6084	−0.3909	0.5117
QLD1	*p*-value	1.0000	1.0000	1.0000	1.0000	1.0000	1.0000	1.0000	1.0000
QLD2	Statistics	−2.2111	1.0125	0.7260	2.7675	2.1013	0.1648	0.2215	3.1511^**^
QLD2	*p*-value	0.4865	1.0000	1.0000	0.1017	0.6410	1.0000	1.0000	**0.0293**
QLD3	Statistics	−0.6188	−1.0859	−1.4378	1.8802	1.8277	0.4982	−0.0048	−0.6831
QLD3	*p*-value	1.0000	1.0000	1.0000	1.0000	1.0000	1.0000	1.0000	1.0000
SA1	Statistics	1.2517	0.3504	−0.3402	2.9022*	−0.7021	0.7780	0.4911	1.0832
SA1	*p*-value	1.0000	1.0000	1.0000	**0.0667**	1.0000	1.0000	1.0000	1.0000
TAS1	Statistics	0.7128	1.4346	−0.0736	0.4477	0.1497	2.0318	0.3194	1.3158
TAS1	*p*-value	1.0000	1.0000	1.0000	1.0000	1.0000	0.7592	1.0000	1.0000
VIC1	Statistics	−1.0145	−1.6814	0.3363	0.0000	−0.6754	−1.6906	−0.3425	0.6725
VIC1	*p*-value	1.0000	1.0000	1.0000	1.0000	1.0000	1.0000	1.0000	1.0000
VIC2	Statistics	0.6126	30.6157^***^	29.6603^***^	30.1835^***^	28.5593^***^	20.9707^***^	17.0234^***^	31.5525^***^
VIC2	*p*-value	1.0000	**0.0000**	**0.0000**	**0.0000**	**0.0000**	**0.0000**	**0.0000**	**0.0000**
VIC3	Statistics	1.1924	18.7642^***^	17.5880^***^	17.5623^***^	16.7609^***^	9.3049^***^	7.2829^***^	19.7776^***^
VIC3	*p*-value	1.0000	**0.0000**	**0.0000**	**0.0000**	**0.0000**	**0.0000**	**0.0000**	**0.0000**
WA1	Statistics	4.6269^***^	3.2538^**^	1.3965	3.1902^**^	1.7224	1.6745	0.3430	6.8012^***^
WA1	*p*-value	**0.0001**	**0.0205**	1.0000	**0.0256**	1.0000	1.0000	1.0000	**0.0000**
WA2	Statistics	3.8885^***^	1.3583	0.3939	−0.1568	0.5833	1.6344	2.7819^*^	3.4730^***^
WA2	*p*-value	**0.0018**	1.0000	1.0000	1.0000	1.0000	1.0000	**0.0973**	**0.0093**

*, **, *** *mean significant at p < 0.1, p < 0.05, and p < 0.01, respectively. The bold values are statistically significant values*.

**Table 7:** Dunn tests on last day of control.

**Last Day of Control**		**Comparisons between Control Strategies 2 to 9 and Control Strategy 1 (stamping out only)**							
**Incursion scenario**	**Dunn test statistics**	**1 vs. 2**	**1 vs. 3**	**1 vs. 4**	**1 vs. 5**	**1 vs. 6**	**1 vs. 7**	**1 vs. 8**	**1 vs. 9**
NSW1	Statistics	−8.9822^***^	−0.6765	−0.1648	−0.6704	−1.3076	0.4781	−1.0602	−10.8082^***^
NSW1	*p*-value	**0.0000**	1.0000	1.0000	1.0000	1.0000	1.0000	1.0000	**0.0000**
NSW2	Statistics	0.3989	2.1061	0.0786	0.9690	0.3778	1.2704	1.0085	−0.3777
NSW2	*p*-value	1.0000	0.6335	1.0000	1.0000	1.0000	1.0000	1.0000	1.0000
NSW3	Statistics	−0.2852	−9.1792^***^	−1.0577	−7.8178^***^	−3.2603^**^	−0.7075	−0.6496	−8.9966^***^
NSW3	*p*-value	1.0000	**0.0000**	1.0000	**0.0000**	**0.0200**	1.0000	1.0000	**0.0000**
QLD1	Statistics	−2.5167	−2.6580	−0.7841	−2.1815	−2.7051	−2.0661	−1.8084	−2.9727^*^
QLD1	*p*-value	0.2132	0.1415	1.0000	0.5246	0.1229	0.6987	1.0000	**0.0531**
QLD2	Statistics	−3.3983^**^	−8.5038^***^	−6.4720^***^	−8.3998^***^	−7.4976^***^	−5.9211^***^	−1.0667	−10.1058^***^
QLD2	*p*-value	**0.0122**	**0.0000**	**0.0000**	**0.0000**	**0.0000**	**0.0000**	1.0000	**0.0000**
QLD3	Statistics	−0.6267	−1.6059	−1.7620	0.7397	0.8837	0.0145	−1.2479	−0.8343
QLD3	*p*-value	1.0000	1.0000	1.0000	1.0000	1.0000	1.0000	1.0000	1.0000
SA1	Statistics	1.1272	−0.6250	−1.4629	1.4540	−1.0461	0.8012	0.6988	0.8665
SA1	*p*-value	1.0000	1.0000	1.0000	1.0000	1.0000	1.0000	1.0000	1.0000
TAS1	Statistics	0.4497	−0.5745	−0.3701	−0.4942	−0.5315	0.4408	−0.1987	0.3866
TAS1	*p*-value	1.0000	1.0000	1.0000	1.0000	1.0000	1.0000	1.0000	1.0000
VIC1	Statistics	−1.9164	−1.9033	0.0000	−0.3165	−0.9533	−1.9104	−0.3243	−1.2788
VIC1	*p*-value	0.9957	1.0000	1.0000	1.0000	1.0000	1.0000	1.0000	1.0000
VIC2	Statistics	0.4837	30.5759^***^	28.9434^***^	30.6717^***^	26.7298^***^	22.9713^***^	16.4683^***^	29.3161^***^
VIC2	*p*-value	1.0000	**0.0000**	**0.0000**	**0.0000**	**0.0000**	**0.0000**	**0.0000**	**0.0000**
VIC3	Statistics	0.9270	25.4775^***^	25.7184^***^	24.7720^***^	23.4256^***^	12.2731^***^	7.9626^***^	22.7966^***^
VIC3	*p*-value	1.0000	**0.0000**	**0.0000**	**0.0000**	**0.0000**	**0.0000**	**0.0000**	**0.0000**
WA1	Statistics	1.3173	−4.5509^***^	2.4955	−5.6252^***^	−1.8958	2.7270	1.8492	−5.3097^***^
WA1	*p*-value	1.0000	**0.0001**	0.2264	**0.0000**	1.0000	0.1150	1.0000	**0.0000**
WA2	Statistics	1.0975	−5.4508^***^	−0.1338	−5.9318^***^	−6.8597^***^	0.6469	0.6700	−5.4624^***^
WA2	*p*-value	1.0000	**0.0000**	1.0000	**0.0000**	**0.0000**	1.0000	1.0000	**0.0000**

*, **, *** *mean significant at p < 0.1, p < 0.05, and p < 0.01, respectively. The bold values are statistically significant values*.

### Alternative Vaccination Strategies and the Size and Duration of Large Outbreaks

**Figure 3:**
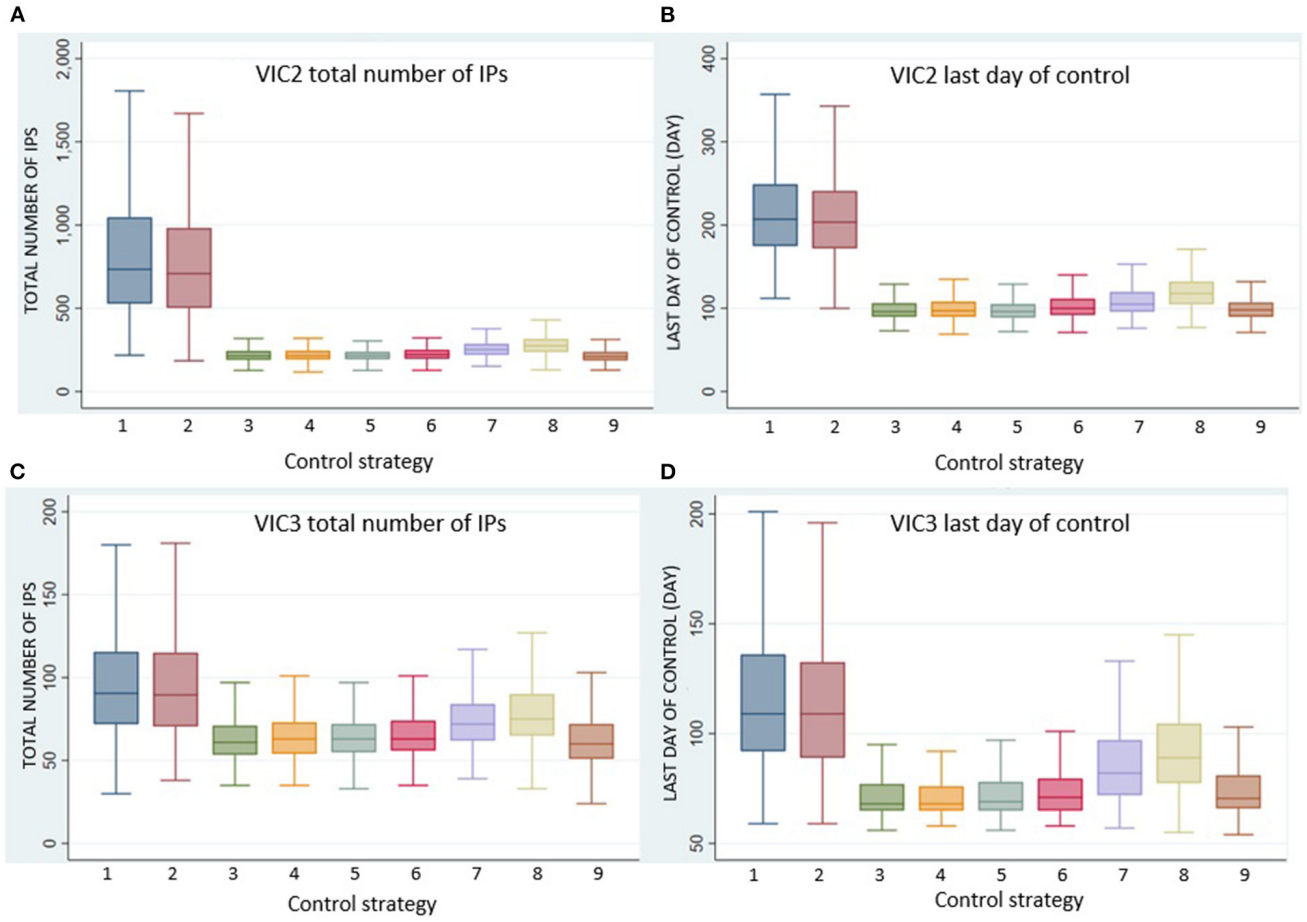
Boxplots of **(A)** VIC2 total number of IPs, **(B)** VIC2 last day of control, **(C)** VIC3 total number of IPs, and **(D)** VIC3 last day of control for each control strategy, Control Strategy 1 to Control Strategy 9.

Here, we focused on comparing the seven alternative vaccination approaches (Control Strategy 3 to Control Strategy 9) with the benchmark stamping out approaches (Control Strategy 1 and Control Strategy 2) for the two incursion scenarios in Victoria, VIC2 and VIC3, which were associated with larger outbreak sizes and for which vaccination was shown to be very effective in reducing size and duration of the outbreaks. VIC2 begins in a dairy herd in southwest Victoria, and VIC3 begins in an intensive beef property in southeast Victoria (see Table 2 and Figure 1). Figure 3 compares the effect of the different vaccination strategies on outbreak size and duration.

**Figure 4:**
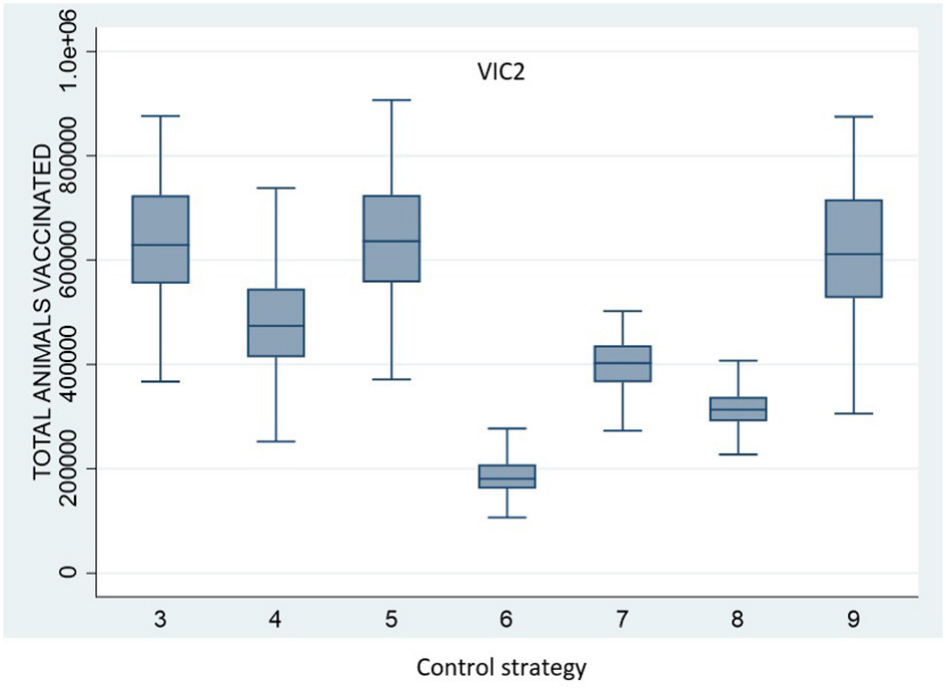
Boxplots of the total number of vaccinated animals for Scenario VIC2 for the control strategies with vaccination (Control Strategy 3 to 9).

**Figure 5:**
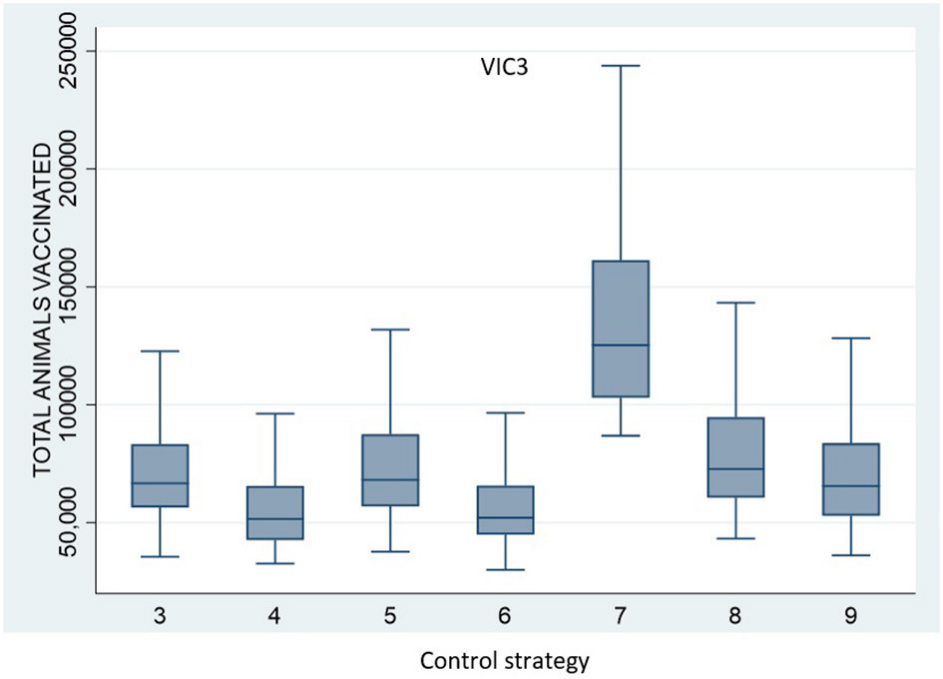
Boxplots of the total number of vaccinated animals for Scenario VIC3 for the control strategies with vaccination (Control Strategy 3 to 9).

All vaccination strategies were effective in reducing outbreak size and duration. However, Control Strategy 7 and Control Strategy 8 (the annulus strategies) were less effective than the ring vaccination strategies. Additionally, there was little difference in outbreak size and duration for Control Strategies 3, 4, 5, and 6. There were significant differences, however, in the numbers of animals vaccinated under the different strategies. The total number of vaccinated animals is shown for each vaccination strategy for scenario VIC2 in Figure 4 and for scenario VIC3 in Figure 5. Tables 8, 9 present the results of the Dunn test of statistical differences comparing these strategies.

**Table 8:** Dunn test of total animals vaccinated for Scenario VIC2.

**Control strategies**	**Dunn test statistics**	**Control strategies**					
		**3**	**4**	**5**	**6**	**7**	**8**
4	Statistics	**12.8766**					
	*p*-value	0.0000^***^					
5	Statistics	−0.08046	**−1.30E + 01**				
	*p*-value	1.0000	0.0000^***^				
6	Statistics	**38.0533**	**25.1767**	**38.1338**			
	*p*-value	0.0000^***^	0.0000^***^	0.0000^***^			
7	Statistics	**20.6108**	**7.7342**	**20.6912**	**−1.74E + 01**		
	*p*-value	0.0000^***^	0.0000^***^	0.0000^***^	0.0000^***^		
8	Statistics	**29.3102**	**16.4336**	**29.3907**	**−8.7431**	**8.6995**	
	*p*-value	0.0000^***^	0.0000^***^	0.0000^***^	0.0000^***^	0.0000^***^	
9	Statistics	1.6069	**−1.13E + 01**	1.6873	**−3.64E + 01**	**−1.90E + 01**	**−2.77E + 01**
	*p*-value	1.0000	0.0000^***^	0.9612	0.0000^***^	0.0000^***^	0.0000^***^

*, **, *** *mean significant at p < 0.1, p < 0.05, and p < 0.01, respectively. The bold values are statistically significant values*.

**Table 9:** Dunn test of total animals vaccinated for Scenario VIC3.

**Control strategies**	**Dunn test statistics**	**Control strategies**					
		**3**	**4**	**5**	**6**	**7**	**8**
4	Statistics	**10.8898**					
	*p*-value	0.0000^***^					
5	Statistics	−1.0787	**−1.20E + 01**				
	*p*-value	1.0000	0.0000^***^				
6	Statistics	**10.1952**	−0.6946	**11.2739**			
	*p*-value	0.0000^***^	1.0000	0.0000^***^			
7	Statistics	**−2.12E + 01**	**−3.21E + 01**	**−2.01E + 01**	**−3.14E + 01**		
	*p*-value	0.0000^***^	0.0000^***^	0.0000^***^	0.0000^***^	
8	Statistics	**−4.3208**	**−1.52E + 01**	**−3.2421**	**−1.45E + 01**	**16.8406**	
	*p*-value	0.0002^***^	0.0000^***^	0.0125**	0.0000^***^	0.0000^***^	
9	Statistics	1.5089	**−9.3809**	2.5876	**−8.6863**	**22.6704**	**5.8298**
	*p*-value	1.0000	0.0000^***^	0.1015	0.0000^***^	0.0000^***^	0.0000^***^

*, **, *** *mean significant at p < 0.1, p < 0.05, and p < 0.01, respectively. The bold values are statistically significant values*.

Control Strategy 6 consistently performed well in this study. This strategy applies vaccination to specialist cattle producers within a 5-km radius around each IP, including feedlots and dairy and intensive beef farms, but excluding mixed beef–sheep farms to avoid including large numbers of sheep on mixed farms in the vaccination program. Note that for Control Strategy 8, very few farms met the stringent criteria to be vaccinated. For example, under scenario VIC2, the median number of premises being vaccinated per run was only 1 (range 0–8).

**Figure 6:**
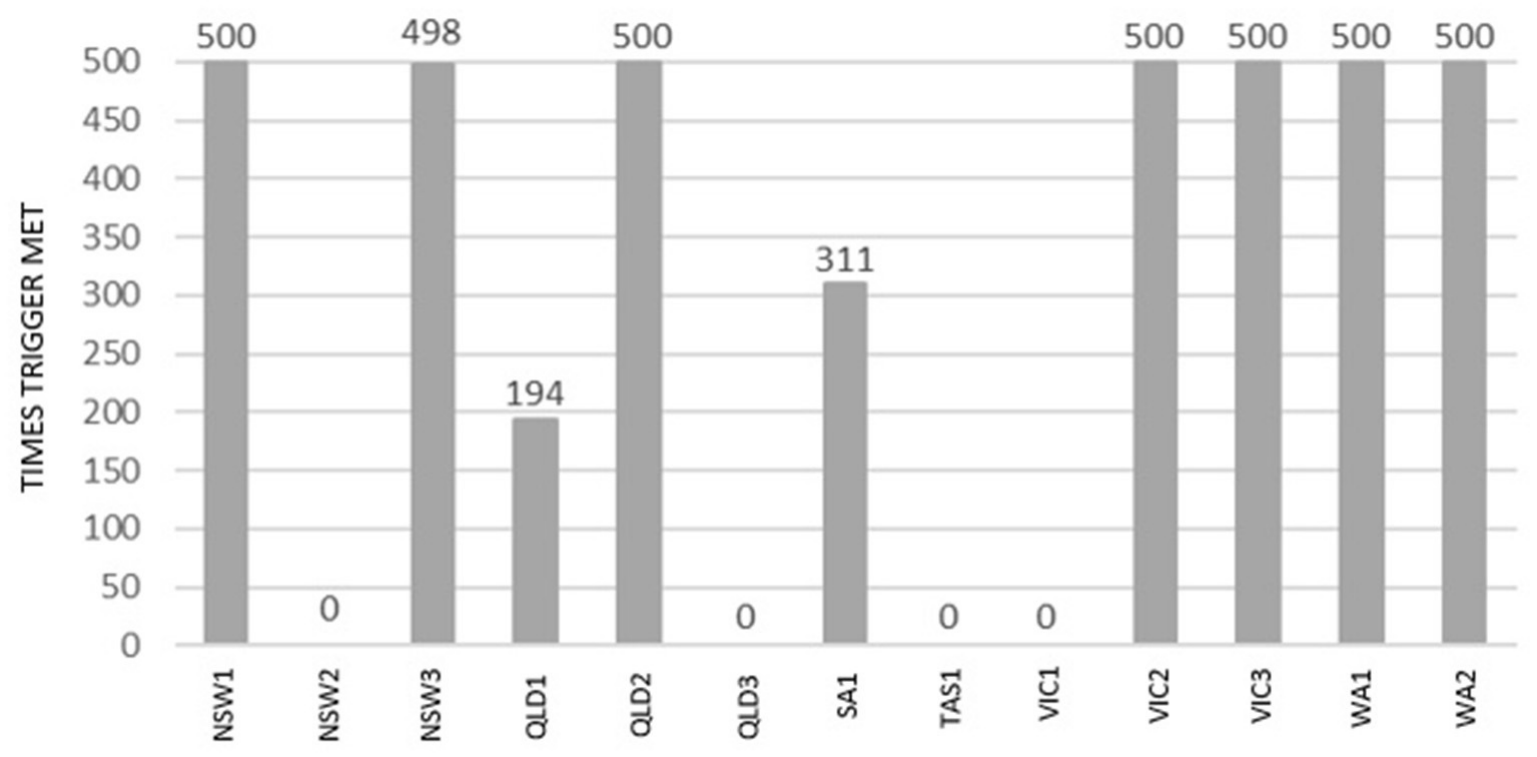
Number of simulations the vaccination trigger was met during 500 simulations of Control Strategy 3 for each incursion scenario.

Figure 6 presents the proportions of the simulation runs for each incursion scenario where the vaccination trigger of five or more infected premises on day 14 of the control phase was met (out of 500 simulations runs for Control Strategy 3).

It is worth noting that for Control Strategy 3, vaccination was triggered in 7 of the 13 scenarios (NSW1, NSW3, QLD3, VIC2, VIC3, WA1, and WA2). On the other hand, there were four scenarios where vaccination was never triggered (NSW2, QLD3, TAS1, and VIC1). Vaccination was never triggered in these scenarios because there were fewer than five IPs on Day 14 of the control phase.

### Results of Sensitivity Analyses

Sensitivity analysis around the assumptions of the timing of vaccination (day 10 vs. day 21 vs. baseline day 14) and vaccination ring radius (3 km vs. baseline 5 km) suggests that results are robust to changes in the assumptions around vaccination. Table 10 presents the Dunn test results for the assumptions of the timing of vaccination (day 10 vs. day 21) and vaccination ring radius (3 km vs. baseline 5 km).

**Table 10:** Dunn tests for sensitivity analyses—comparisons of Control Strategy 1 with Control Strategy 3 for baseline simulation assumptions and changed vaccination assumptions for sensitivity analysis.

**Variable**	**Incursion scenario**	**Dunn test statistics**	**Baseline simulations**	**Sensitivity analysis simulations with changed assumptions**		
			**(B)**	**(1)**	**(2)**	**(3)**
			**Day 14/5 km**	**Day 14/3 km**	**Day 10/5 km**	**Day 21/5 km**
			**Control Strategy 1 vs. 3**	**Control Strategy 1 vs. 3**	**Control Strategy 1 vs. 3**	**Control Strategy 1 vs. 3**
Number of IPs	VIC2	Statistics	31.5108	20.6578	24.5240	23.0540
	VIC2	*p*-value	0.0000^***^	0.0000^***^	0.0000^***^	0.0000^***^
Last day of control	VIC2	Statistics	31.1772	18.4195	23.8080	27.0068
	VIC2	*p*-value	0.0000^***^	0.0000^***^	0.0000^***^	0.0000^***^
Number of IPs	VIC3	Statistics	25.0053	22.3874	20.7088	18.4084
	VIC3	*p*-value	0.0000^***^	0.0000^***^	0.0000^***^	0.0000^***^
Last day of control	VIC3	Statistics	18.3969	16.5565	17.4394	12.4666
	VIC3	*p*-value	0.0000^***^	0.0000^***^	0.0000^***^	0.0000^***^

*(B) Baseline assumption: vaccination from Day 14 and 5-km vaccination ring radius*.

*(1) Alternative: vaccination from Day 14 and 3-km vaccination ring radius*.

*(2) Alternative: vaccination from Day 10 and 5-km vaccination ring radius*.

*(3) Alternative: vaccination from Day 21 and 5-km vaccination ring radius*.

*, **, *** *mean significant at p < 0.1, p < 0.05, and p < 0.01, respectively*.

## Discussion and Conclusion

This paper shows the results of a simulation study informed by stakeholder consultation that investigated options for incorporating vaccination into control strategies for FMD outbreaks across Australia, including areas considered to be at lower risk for introduction and spread of FMD.

For previously FMD-free countries, FMD control has tended to be based on stamping out and indeed this is Australia's preferred approach as described in AUSVETPLAN (3). However, the use of vaccination in control of an FMD outbreak is increasingly recognized as an important option (9, 25, 26). This is driven by resourcing issues and ethical, environmental, and welfare concerns over the large-scale culling of animals (5, 25, 27–30). While vaccination may contribute to earlier eradication of the disease, it will be associated with additional costs—keeping vaccinated animals in the population will delay the period until FMD-free status is regained under current World Organization for Animal Health standards (13, 31) and add additional complexity to post-outbreak surveillance programs (32).

This analysis has shown that many outbreaks of FMD in Australia, based on incursion scenarios identified by stakeholders, were comparatively small. Management through stamping out without vaccination may be the most appropriate response for these smaller outbreaks, as vaccination did not reduce the size or duration and the cost of vaccination may increase control costs substantially. The largest simulated outbreaks were observed for two Victorian incursion scenarios (VIC2 and VIC3). This is consistent with previous work that identified southeastern Australia as the area most vulnerable to an FMD outbreak because of its geographic and climatic conditions, including its relatively high human population and its higher stocking rates (20). In Victoria, the temperature, climate, and higher rainfall mean that there is more intensive farming than in most other parts of Australia.

For the large simulated outbreaks in Victoria, vaccination was shown to reduce both the size (total number of IPs) and length of an outbreak. This finding is also consistent with previous modeling studies in Australia (4, 5, 28) and overseas (9, 25, 26, 33–36), which found that vaccination can be an effective strategy in suppressing the spread of infection particularly if livestock density is high, disease is widespread, there is a high rate of spread, or resources for stamping out are limited.

Suppressive ring vaccination, that is, vaccinating in a ring immediately around IPs, was found to be more effective than vaccination in an annulus, further out from the IPs. A previous study using multiple models and a United Kingdom outbreak scenario also concluded that suppressive ring vaccination was a more effective use of vaccine resources (5). A similar impact on outbreak size and duration was found regardless of the approach to ring vaccination (Control Strategies 3–6). However, there were significant differences in the numbers of animals vaccinated under the different strategies. Vaccinating cattle only was particularly effective. In their multi-model study, Roche et al. (9) reported that a cattle-only vaccination strategy was as effective as vaccinating all susceptible species for three of the four models used in their study.

Issues with management of vaccinated animals following an FMD outbreak and trade restrictions have limited the use of vaccination as a first-line control strategy, especially for countries with large export industries. FMD-free status can be recovered 3 months after the last reported case under stamping-out or pre-emptive culling strategies, and this increases to 6 months when vaccination is used unless all vaccinated animals are removed from the population, in which case free status can be regained 3 months after removing the vaccinated animals (13). To minimize duration of the closure of export markets, under current international standards, vaccinated animals would need to be removed from the population (31). However, culling vaccinated animals obviously has additional animal welfare, economic, and social impacts. In this situation, it would be desirable to minimize the number of animals vaccinated while still achieving effective control. This study and others [e.g., (9)] confirm that selective, targeted vaccination can be an effective strategy to reduce the number of animals vaccinated. We found that targeting vaccination to high-risk areas (strategy Control Strategy 4) or to cattle only (Control Strategy 6) achieved effective control of the large Victorian outbreak scenarios, while significantly reducing the number of animals vaccinated compared to more expansive vaccination strategies.

Given the finding that vaccination when used with stamping can be very effective in reducing the size and duration of large outbreaks compared to stamping out on its own, a key issue is deciding when it should be used. That is, how can decision makers identify situations when an outbreak is likely to be large. A decision to vaccinate early in the outbreak may result in situations where it was not actually required and have consequent implications for post-outbreak surveillance, management of vaccinated animals, and regaining FMD-free status and access to export markets (31, 32). Conversely, not using vaccination in some situations may lead to much larger and longer outbreaks, increased control costs, and greater impacts on industry and local communities (6). During an outbreak, decisions on control are often made under significant uncertainty and in conditions that are continually evolving. Resources are often limited and will influence the effectiveness of disease control efforts. The decision to vaccinate and choice of strategy will ultimately depend on the nature of the epidemic, available resources to implement it, and objectives of the control program [(37); also see AUSVETPLAN, (3)]. Work by Hutber et al. (38), Halasa et al. (39), and Sarandopoulos (40) indicates that information available early in an outbreak can be used to make inferences about the potential severity of an FMD outbreak. In a detailed study involving simulated FMD outbreaks in Australian and New Zealand, Garner et al. (6) showed that relatively simple metrics that would be available to disease managers early in an outbreak such as the cumulative number of IPs were consistently found to be strongly associated with the final size and the duration of the outbreak.

There are two key implications from these findings. First, combining stakeholder consultation to formulate scenarios and strategies for epidemiological modeling revealed that many incursion scenarios of concern to stakeholders in Australia are likely to lead to small outbreaks. These outbreaks could be managed effectively with stamping out alone and is consistent with findings in other low livestock density situations (33). This highlights the importance of incorporating the views and expertise of stakeholders in scenario formulation and not just focusing on large, worst-case scenarios when comparing control strategies. Stakeholder consultation helped identify the concerns and priorities of disease managers across the Australian jurisdictions and ensured that the simulations were driven by decision-makers' needs rather than just the possibilities of the modeling platform.

Second, notwithstanding the effectiveness of vaccination to reduce the size and duration of large outbreaks, under current international standards (13), there remains a strong disincentive to use vaccination under the belief that a vaccination policy will always result in the longest return to markets for exports of susceptible livestock and their products. To minimize trade impacts, vaccinated animals need to be removed from the population at the end of the outbreak. Given this situation, we have shown that targeted vaccination strategies are effective in achieving control while reducing the numbers of animals vaccinated. Differential time periods are being challenged (41) and new diagnostic approaches that improve surveillance might be able to provide acceptable levels of confidence in the infection status of vaccinated populations in the future.

Future research could further investigate and validate the effectiveness of vaccination as a control strategy for FMD. We suggest analysis to determine whether vaccination can reduce the probability of extremely large and long outbreaks. In this study, the focus was on the median size and duration of an outbreak. Examining the effect of alternative control strategies on the probability of large and long outbreaks will provide decision makers with a better understanding of the potential role of vaccination. An additional area for further work also includes spatially and temporally mapping the risk of FMD spread to help identify regions where vaccination is more likely to play a useful role. More comprehensive modeling studies could be used to assess which areas may be more vulnerable or susceptible to large outbreaks. Further work to refine early decision indicators of severe outbreaks to support decision-making is important. Lastly, we recommend further research to investigate the trade-offs between the cost of using vaccination as a control strategy and the effectiveness of the outcome. The costs should include consideration of direct costs of the control strategies and indirect costs, such as revenue loss from animal movement restrictions, loss from trade embargoes, and the cost of business recovery and continuity after eradication. The effectiveness of the outcome should be considered not only in terms of infected premises and control duration but also in terms of numbers of animals vaccinated and culled. This could consider the ethical, welfare, and social benefits of reducing culling using vaccination.

## Data Availability Statement

The raw data supporting the conclusions of this article will be made available by the authors, without undue reservation.

## Ethics Statement

The studies involving human participants were reviewed and approved by CSIRO Human Ethics research committee. The patients/participants provided their written informed consent to participate in this study.

## Author Contributions

TC: project lead and main author of manuscript writing. MG: epidemiological modeling and write up of methods and results. ST: statistical analysis and write up of methods and results. SR and SL: stakeholder consultation design and implementation. CM, ACB, and SH: provided input toward conceptual model and analysis of results. All authors contributed to the article and approved the submitted version.

## Conflict of Interest

The authors declare that the research was conducted in the absence of any commercial or financial relationships that could be construed as a potential conflict of interest.

## Publisher's Note

All claims expressed in this article are solely those of the authors and do not necessarily represent those of their affiliated organizations, or those of the publisher, the editors and the reviewers. Any product that may be evaluated in this article, or claim that may be made by its manufacturer, is not guaranteed or endorsed by the publisher.

## Footnotes

^1^AADIS is available under license for research purposes from the Australian Government Department of Agriculture, Water and the Environment (DAWE). Please contact Andrew.Breed@awe.gov.au at the Epidemiology and One Health Section, DAWE, for more information.
