# Supplementary material for: A Simulation Study of the Use of Vaccination to Control Foot-and-Mouth Disease Outbreaks Across Australia
Source: Front Vet Sci. 2021 Aug 11;8:648003. doi: 10.3389/fvets.2021.648003 (PMC8385296; doi:10.3389/fvets.2021.648003)
Supplement: Supplementary file 1 [file Data_Sheet_1.PDF]

Supplementary Material

I. AADIS - Key model parameters and settings

Table I.1 Population structure

Herd type	Description	N
1	beef extensive	3993
2	beef intensive	51383
3	feedlot	508
4	mixed beef	21556
5	mixed sheep	21556
6	dairy	8675
7	pigs small	1872
8	pigs large	334
9	sheep	22150
10	smallholder	103641
	total	235668

Table I.2 Within-herd EBM parameters

Herd type	beta	Latent period (days)	Infectious period (days)	Clinical period (days)	Days until clinical	Proportion clinical
beef extensive	0.7	2	4	12	5	1
beef intensive	2	2	4	12	5	1
feedlot	8	2	4	12	5	1
mixed beef	2	2	4	12	5	1
mixed sheep	0.8	2	7	5	5	0.5
dairy	6	2	4	12	5	1
pigs small	6	1	4	10	4	1
pigs large	8	1	4	10	4	1
sheep	0.8	2	7	5	5	0.5
smallholder	2	2	5	9	5	0.75

Table I.3 Model settings (disease transmission)

Parameter	Value
database name	AADIS_CSIRO1
scenario name	csiro_V2_SO1
scenario start date mode	fixed
scenario end mode	earliest
scenario fixed length	730
scenario num runs	500
seed mode	snapshot
snapshot seed file	csiro_V2_scenario_end_snapshot_run_29
pre-infection start offset	21
**Direct spread**	
direct spread search distance tolerance 1	10 km
direct spread search distance tolerance 2	100 km
**Indirect Spread**	
indirect spread base probability	0.05
indirect spread min new infections	1
indirect spread mode new infections	2
indirect spread max new infections	5
indirect spread search distance tolerance 1	10 km
indirect spread search distance tolerance 2	100 km
**Local spread**	
local spread base probability	0.036
local spread base multi herd probability	0.5
local spread detection weight	0.66
local spread radius	3 km
local spread min new infections	1
local spread mode new infections	2
local spread max new infections	5
**Airborne spread**	
airborne spread plume distance base	0.113
airborne spread plume distance exp	1.367
airborne spread plume distance max	20 km
airborne spread decay mode	linear
airborne spread min new infections	1
airborne spread mode new infections	2
airborne spread max new infections	5
grid-based weather data	TRUE
**Saleyard spread**	
saleyard spread mode	regular
saleyard spread search distance tolerance 1	20 km
saleyard spread search distance tolerance 2	100 km

Table I.4 Model settings (control)

Parameter/setting	Value
Control measures enabled	TRUE
movement restrictions enabled	TRUE
SP reporting enabled	TRUE
SP false report ratio	2.34
SP moving average	3
SP false report RA ratio	0.6
SP false report CA ratio	0.3
SP false report OA ratio	0.1
direct tracing enabled	TRUE
indirect tracing enabled	TRUE
false traces enabled	TRUE
tracing backwards window	14 days
tracing forwards window	14 days
surveillance enabled	TRUE
surveillance local radius	3 km
surveillance min clinical prevalence	0.05
surveillance min days infected	10
surveillance lab results required	TRUE
surveillance lab results delay	1 day
surveillance overdue DCP SP TP threshold	2
IP operations enabled	TRUE
vaccination enabled	FALSE
vaccination start day	14
vaccination start IP count	0
vaccination immunity lag	6
resources enabled	TRUE

Table I.5 Scenario settings (control)

**Parameter**	**Setting**
Time to detect first infected property (days)	21
National livestock standstill period (days)	3
Control Area (CA) initial size	Whole state/territory
Restricted Area (RA) initial size	Local government area (LGA)
Days into the control program before CA and RA are reduced in size	14 and a further 14
Control Area (CA) subsequent size	Radial zone around IP of 50 km radius, then 10 km radius
Restricted Area (RA) subsequent size	Radial zone around IP of 10 km, then 3 km radius
Vaccination start	14 days into control program
Vaccination zone	ring (5 km) or annulus (5-10 km) around IPs depending on strategy
Species vaccinated	Variable depending on strategy

Table I.6 Resources for control

**Jurisdiction**	**Surveillance teams**		**Culling teams**		**Disposal teams**		**Disinfection teams**		**Vaccination teams**	
	initial	max	initial	max	initial	max	initial	max	initial	max
NSW	5	60	2	15	3	20	10	100	20	500
Vic	3	50	1	13	3	20	10	100	12	500
Qld	5	40	1	13	2	14	8	100	20	500
SA	5	20	1	8	2	10	7	100	15	500
WA	4	30	1	10	2	11	5	100	10	500
Tas	4	15	1	5	2	7	5	100	15	500
NT	2	15	1	5	2	8	5	100	5	500
Australia	17	138	5	41	9	54	30	420	58	2100

II. Stakeholder consultation survey questions

Table II.1 Questions from stakeholder workshops

1. Name?
2. What is your day-to-day area of responsibility?
3. What role would you play during the event of a FMD or other EAD outbreak?
4. What kinds of challenges would you expect to encounter during an outbreak? (I.e. what could go wrong?)
5. What kinds of economic impacts would you expect as the result of an FMD outbreak?<br>6. Which of these are most concerning?
7. What kinds of economic impacts would you expect as the result of the *management response* to an FMD outbreak?<br>8. Which of these are most concerning?
9. What kind of economic considerations do you think would most influence *decision-making process*es during an FMD outbreak?

Table II.2 Questions from expert elicitation on FMD outbreak scenarios and control strategies

**1. What are the most important or relevant scenarios for an outbreak of FMD in your jurisdiction? Please provide a description of 3 scenarios.**					
	**When**	**How**	**What species and farm type and where**	**Time to detect**	**Why is this scenario important or relevant?**
Example	*April*	*Introduction of FMD through contaminated salami*	*Backyard pig farm in greater Melbourne area*	*3 weeks*	
**Scenario 1**					
**Scenario 2**					
**Scenario 3**					

**2. Please list the control strategies that are of most interest or importance to your jurisdiction in the event of an FMD outbreak. Please provide information on the following areas. Please note you can provide more than 3 options.**			
**Control measure**	**Option 1**	**Option 2**	**Option 3**
**Standstill period (days)**<br>Period of time at the start of the outbreak that the national livestock standstill is in force	e.g. 7	e.g. 10	
**Movement control areas (RA and CA) (km)**<br>Area around IP where movement restrictions apply. *You may also want to consider that this area can be reduced in size after a designated period of time. If relevant please list the time period (days) and new area size (km).*	*e.g. RA 3km around IP and CA 5km around IP*	*e.g. RA in the local district area and CA the whole state then after 14 days reduce RA and CA to 3km and 5km respectively*	

**Control strategy**<br>How to manage the outbreak using stamping out (cull IP) plus or minus other strategies such as destruction of DC, pre-emptive destruction on ARP, *etc*. Vaccination to be considered below.	*e.g. stamping out alone*	*e.g. stamping out plus cull DC*	*e.g. stamping out plus pre-emptive ring cull 2km around IP*
**Vaccination control strategy (if vaccination is used)**			
**Vaccination program trigger**<br>The conditions under which a vaccination program is commenced	*e.g. day 14 of the control program*	*e.g. once 10 IP have been declared*	
**Vaccination ring size (km)**<br>Area around IP to vaccinate. These areas can be immediately around IP (so acts to supress spread) or a 'donut' a specified distance from the IP (so acts like a protective buffer and often surrounds the RA). In the latter please indicate the inner and outer ring distance.	*e.g. 3km around IP (inner radius 0km from IP, outer radius 3km)*	*e.g. 2km donut around IP (inner radius 3km from IP, outer radius 5km from IP)*	
**Species and/or farms to vaccinate**<br>For example cattle only, mixed sheep and cattle farms only, dairy farms only, intensive beef and dairy farms, all farms except smallholder farms *etc.*	*e.g. dairy farms only*	*e.g. cattle (excluding feedlots) and sheep only*	*e.g. all farms except smallholders and pigs*
**Order of vaccination**<br>Vaccinate from the outside of the ring to the inside or vice-versa	*e.g. inside-out*	*e.g. inside-out*	
**How to start vaccinating**<br>Whether vaccination rings should only be established around IP declared after the vaccination program has commenced	*e.g. vaccinate around new IP only*	*e.g. vaccinate around ALL IP*	*e.g. vaccinate around new IP and any IP declared in the previous 7 days*
**Area to vaccinate**<br>Vaccinate in high risk areas (high animal densities) only or in all areas	*e.g. high risk areas only*	*e.g. all areas*	
